# Identification of a novel germ-line mutation in the *TP53 *gene in a Mexican family with Li-Fraumeni syndrome

**DOI:** 10.1186/1477-7819-7-97

**Published:** 2009-12-17

**Authors:** Lucia Taja-Chayeb, Silvia Vidal-Millán, Olga Gutiérrez-Hernández, Catalina Trejo-Becerril, Enrique Pérez-Cárdenas, Alma Chávez-Blanco, Erick de la Cruz-Hernández, Alfonso Dueñas-González

**Affiliations:** 1 Instituto Nacional de Cancerología (INCan), Mexico City, México; 2 Unidad de Investigación Biomédica en Cáncer, Instituto de Investigaciones Biomédicas (IIB), Universidad Nacional Autónoma de México (UNAM), Mexico City, México

## Abstract

**Background:**

Germ-line mutations of the *TP53 *gene are known to cause Li-Fraumeni syndrome, an autosomal, dominantly inherited, high-penetrance cancer-predisposition syndrome characterized by the occurrence of a variety of cancers, mainly soft tissue sarcomas, adrenocortical carcinoma, leukemia, breast cancer, and brain tumors.

**Methods:**

Mutation analysis was based on Denaturing high performance liquid chromatography (DHPLC) screening of exons 2-11 of the *TP53 *gene, sequencing, and cloning of DNA obtained from peripheral blood lymphocytes.

**Results:**

We report herein on Li Fraumeni syndrome in a family whose members are carriers of a novel *TP53 *gene mutation at exon 4. The mutation comprises an insertion/duplication of seven nucleotides affecting codon 110 and generating a new nucleotide sequence and a premature stop codon at position 150. With this mutation, the p53 protein that should be translated lacks the majority of the DNA binding domain.

**Conclusion:**

To our knowledge, this specific alteration has not been reported previously, but we believe it is the cause of the Li-Fraumeni syndrome in this family.

## Background

Li-Fraumeni syndrome (LFS) is an autosomal dominantly inherited high-penetrance cancer-predisposition syndrome characterized by the occurrence of a variety of cancers in children and young adults. While the majority of cancer-predisposition syndromes are tissue-specific, such as those associated with breast cancer, colon cancer, and melanoma, LFS is associated with several different cancer types, mainly bone and soft tissue sarcoma, breast cancer, brain tumors, adrenocortical carcinoma, and leukemia [1,2]. These cancers often appear at a young age and can occur several times throughout the life of an affected person. Approximately 70% of LFS families and 8-22% of families with LF like (LFL) carry germ-line mutations at the tumor suppressor gene *TP53 *[3-6]. The majority of missense alterations occur at evolutionarily conserved amino acid residues in the DNA binding domain [7]; outside of this core region, deleterious *TP53 *changes tend to be nonsense or frameshift mutations that cause premature protein-translation termination [8-10]. At present, 399 pathogenic germ-line mutations have been reported for *TP53*, 78% of which are missense mutations principally located at the sequence coding for the DNA binding domain [11]. Epidemiological studies estimate that approximately 70% of males and 100% of females who inherit a *TP53 *mutation are at increased risk for developing cancer of the breast, brain, soft tissue, bone, blood, and adrenal cortex [12].

In order to recognize the syndrome, the French LFS Working Group has developed practical criteria: The so-called Chompret criteria. These criteria integrate the following three different clinical situations suggestive of LFS: (a) a proband with a tumor belonging to the narrow LFS tumor spectrum (soft tissue sarcoma, osteosarcoma, brain tumor, pre-menopausal breast cancer, adrenocortical carcinoma, leukemia, lung bronchioloalveolar carcinoma) prior to the age of 46 years and at least one first- or second-degree relative with LFS tumor (except for breast cancer if the proband is affected by breast cancer) before 56 years of age or with multiple tumors, or (b) a proband with multiple tumors (except multiple breast tumors), two of which belong to the LFS tumor spectrum and the first of which occurred prior to the age of 46 years, or (c) a proband with adrenocortical carcinoma or choroid plexus tumor, irrespective of family history [13,14].

The *TP53 *tumor suppressor gene (chromosome 17p13) encodes a protein that participates in many overlapping cellular pathways that control cell proliferation and homeostasis, such as cell cycle, apoptosis, and DNA repair. The p53 protein is a transcription factor constitutively expressed in the majority of cell types and activated in response to various stress signals (importantly, genotoxic stress) [15]. Loss of p53 function is thought to suppress a mechanism of protection against the accumulation of genetic alterations, as the mutant p53 protein is unable to carry out, i.e., transcriptional transactivation of downstream target genes that regulate the cell cycle and apoptosis. Somatic *TP53 *genetic alterations are found frequently in a variety of human sporadic cancers, with frequencies varying from 10-60%, depending on tumor type or population group [16,17].

In this work, we describe a family with LFS syndrome with one novel *TP53 *germ-line mutation that corresponds to a 7 nucleotide insertion at exon 4, which generates a frameshift and a premature stop codon at position 150. Initially, the mutation was identified in a patient with breast cancer and was based on the pedigree from which the mutation derived from the paternal side, which was corroborated afterward. The mutation was also identified in one other family member (healthy at the moment of the study). These findings bear important implications for genetic counseling and possibly clinical management.

## Patients and Methods

### Family

The family studied is of Mexican origin. The index case was a 23-year-old female diagnosed with breast carcinoma of the left breast with combined histological features of lobular carcinoma and infiltrating ductal carcinoma. The family history suggested LFS: the patient's father was diagnosed with dorsal soft tissue leiomyosarcoma at the age of 67 years, and a half-sister (from the paternal side) died of bronchioloalveolar carcinoma at the age of 25 years. The patient's grandparents died of different causes, but none had cancer. These data were confirmed by clinical files and histopathological reports. Before the molecular analysis, the family received genetic counseling and signed informed consent. This protocol was approved by the local Ethical and Scientific Committees.

### DNA extraction

DNA was obtained from 10 ml of peripheral blood leukocytes. Genomic DNA was extracted with the extraction kit Wizard Genomic DNA purification kit (Promega, Madison, WI, USA), according to manufacturer instructions. DNAs were quantified spectrophotometrically and stored at -20°C.

### Polymerase chain reaction

The oligonucleotides were designed to amplify the coding regions as well as the adjacent intronic sequences. Seven pairs of primers were used to amplify the entire *TP53 *gene as described by Loyant in 2005 [18]. The primer sequence for exon 4 was the following: Forward 5'GGT CCT CTG ACT GCT CTT TTC ACC-3', Reverse 5'-CAG GCA TTG AAG TCT CAT GGA AG-3'. The Polymerase chain reaction (PCR) for Denaturing high performance liquid chromatography (DHPLC) and sequence analysis were performed in a total volume of 25 μl containing 50 ng of DNA, 1 μmol/L of each primer (forward and reverse), 200 μmol/L dNTPs (Applied Biosystems, Foster City, CA, USA), 0.25 U Taq polymerase (Applied Biosystems™), and buffer 1× provided by the manufacturer. PCRs were performed in a 2400 Thermal Cycler (Applied Biosystems). Amplifications were performed according to a touchdown protocol with initial denaturation at 95°C for 5 min and final extension at 72°C for 5 min, denaturation at 95°C for 30 sec, annealing at 56.5-49.5°C and decreasing 0.5°C per cycle for 14 cycles, followed by 16 cycles at 49.5°C; extension carried out at 72°C for 40 sec. Amplification was verified by gel electrophoresis.

### DHPLC

After corroborating a correct amplification, PCR reactions were denatured at 95°C for 10 min and renatured by decreasing the temperature at a rate of 2°C per min to 25°C. Samples were analyzed in a DHPLC device (Transgenomics, Inc., San Jose, CA, USA) according to temperature and elution conditions calculated by DHPLC software. Approximate time of analysis was 9 min per sample. Heterozygote profiles were identified by visual analysis of the chromatograms, comparing peak shapes with a wild-type sample.

### Sequencing

Samples were sequenced to identify sequence change in samples with an aberrant DHPLC profile. PCR amplicons were purified utilizing isopropanol precipitation and then sequenced in both forward and reverse directions, from at least two independent amplification products. Purified DNA was diluted and cycle-sequenced employing the ABI BigDye Terminator kit v3.1 (ABI, Foster City, CA, USA) according to manufacturer instructions. Sequencing reactions were electrophoresed on an ABI3100 genetic analyzer. Electropherograms were analyzed in both sense and antisense direction for presence of mutations. The sequences obtained were compared with the reference *TP53 *(GenBank X54156). Results were compared with the following three databases: International Agency for Research on Cancer (IARC) database; the Human Gene Mutation Database (HGMD), and the *P53 *Knowledgebase [11,19,20].

### Cloning

To determine the precise site and sequence of the insertion, PCR products corresponding to exon 4 were cloned using the TOPO-TA Cloning Kit for Sequencing, (Invitrogen) according to manufacturer instructions. The product of the ligation reaction was transformed into *Escherichia coli *DH5α by calcium precipitation and plated on Luria Bertani-agar plates and selected with 100 μg/ml of ampicillin. Twelve colonies were picked and grown in Luria Bertani media in the presence of ampicillin. Plasmid extraction was performed by the alkaline lysis method and purified for sequencing.

## Results

### *TP53 *Analysis

**Figure 1:**
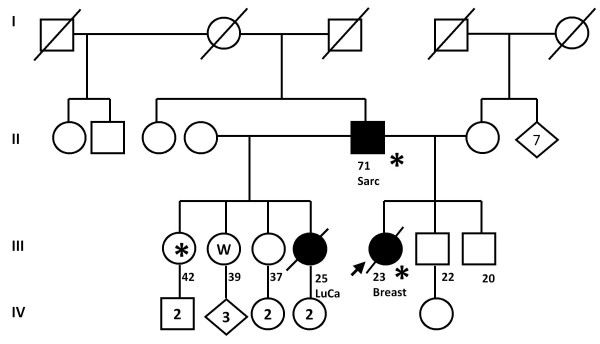
**Pedigree of the studied family**. Square symbols indicate males, round symbols indicate females, diamond symbols indicate individuals of unknown sex, line through symbol means deceased individual. Tumor type and age at diagnosis of the tumors are indicated below the individual identifiers. Sarc = sarcoma; LuC = lung cancer; Breast = breast cancer. * = mutation present; W = mutation absent.

**Figure 2:**
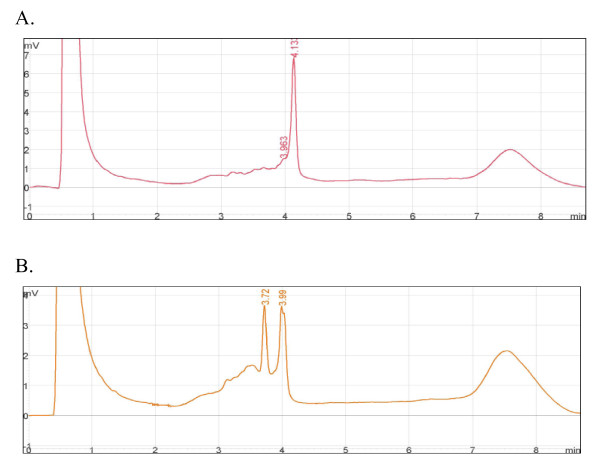
**Denaturing high performance liquid chromatography (DHPLC) analysis of *TP53 *exon 4**. **A**. Wild-type chromatogram profile. **B**. Abnormal elution profile found for the proband. The X axis represent time of elution (retention time), while the Y axis indicates height of the peaks.

**Figure 3:**
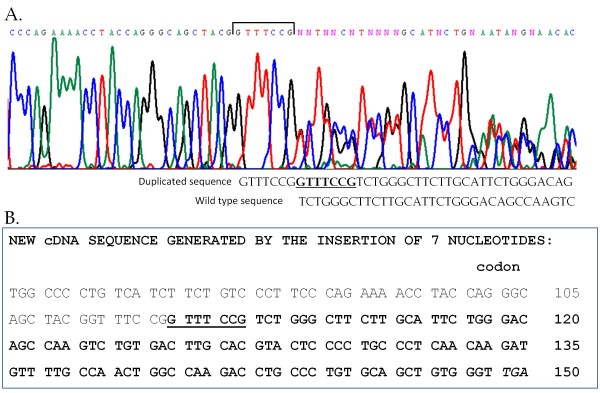
**DNA sequence showing the insertion and cDNA sequence generated by the mutation**. **A**. Sequence analysis of *TP53 *exon 4 for the index patient. Bold underlined indicates the duplicated sequence, below which the wild-type sequence is shown. **B**. Changes in the cDNA sequence: new cDNA sequence (bold) and generation of a premature stop codon (italics). The 7 nucleotide insertion is underlined.

Based on clinical data, the family was diagnosed with LFS. The pedigree (Figure 1) suggested that individual II-5 might be the carrier of the *de novo TP53 *mutation because none of his ancestors had had cancer. The first family member studied was the index patient. We analyzed exons 2-11 by DHPLC and observed an abnormal chromatogram in exon 4 (Figure 2). Subsequent direct sequencing of the aberrant PCR product of exon 4 demonstrated a 7-nucleotide insertion. Further analysis of the sequence demonstrated that the insertion was, in fact, tandem duplication (c.329-330insGTTTCCG). This duplication generates a frameshift and a premature stop signal at codon 150 (Figure 3).

Once the mutation was detected, we searched for the specific alteration in the index patient's father and two half-sisters (paternal side). This mutation was also detected in the father, as well as in patient III-1 (III-2 was wild-type). Additionally, we found that the proband and the two remaining mutated members were homozygous for the Arg >Pro polymorphism at codon 72. We must continue to seek the mutation in other family members; however, they have refused to participate in the study until the present.

### Cloning of the mutated exon 4

**Figure 4:**
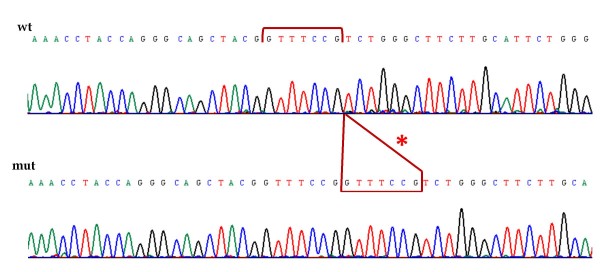
**Sequence of the cloned fragments**. The upper sequence corresponds to the wild-type allele; the red bracket indicates the 7 nucleotide sequence that is duplicated. The lower sequence corresponds to the mutated allele, and the asterisk and red box indicates the inserted/duplicated sequence.

To further characterize the mutation, we cloned the mutated PCR product into the pCR4-TOPO vector (TOPO-TA Cloning Kit for Sequencing, Invitrogen). We sequenced 12 clones and found the mutated allele in five clones. Sequence analysis revealed that the insertion indeed corresponded to GTTTCCG, disrupting codon 110, and inducing a frameshift and generation of a premature stop codon at position 150 (Figure 4). The GTTTCCG sequence corresponds to a duplication of the upstream GTTTCCG sequence.

We searched for this mutation in three different databases: IARC database; *P53 *Knowledgebase, and HGMD [11,19,20] and found that this specific alteration has not been reported either as a germ-line or as somatic mutation.

## Discussion

Identification of a germ-line *TP53 *mutation in a patient allows for the following a) to confirm the diagnosis of LFS on a molecular basis; b) to ensure regular clinical surveillance by an informed clinician in order to avoid a delay in diagnosis of a second tumor; c) to avoid radiation whenever possible, and d) to offer genetic counseling and prenatal diagnosis to the families [21].

In this work, we presented the mutational analysis of a family with LFS in whom the tumor spectra reported, which suggested that family members could have LFS. Molecular analysis revealed an insertion/duplication of 7 nucleotides at exon 4. The mutation was detected in two affected relatives and in one healthy member. The GTTTCCG sequence was duplicated and inserted after the last G. This insertion disrupted codon 110 and generated a shift in the open reading frame and a stop codon at position 150. This alteration should induce the generation of a shorter p53 protein with a different amino acid sequence in its carboxy terminal portion. This means that the DNA binding domain, oligomerization domain, and nuclear localization signals should be lost.

Acquisition of *TP53 *mutations can have two consequences: 1) a dominant negative effect by hetero-oligomerization of the more stable mutant *p53 *with wild-type *p53 *molecules expressed from the normal remaining allele, and 2) a gain of function of the mutant p53 protein [22]. Thus, mutation of *TP53 *may provide a selective advantage for clonal expansion of pre-neoplastic or neoplastic cells. However, all mutations are not equivalent.

Mutant proteins differ in terms of the extent of their loss of suppressor function and by their capacity to inhibit wild-type p53 in a dominant-negative manner. In addition, some p53 mutants apparently exert an oncogenic activity of their own [9]. The *TP53 *region that most frequently contains deletions or insertions is that of codons 151-159: CGC CCG CGC CGC ACC CGC GTC CGC GCC. In fact, approximately 9% of all deletions and insertions and 1% of all *TP53 *mutations have been reported at this G:C-rich sequence with multiple runs and direct repeats [23]. The mutation reported in this work is located before this region.

To our knowledge, this mutation has not been previously reported; however, the mutation site has been reported as involved in several other alterations, including deletions and insertions of one or several nucleotides. In 1994, Birch *et al *[3] reported a complex mutation involving deletion of 11 base pairs and insertion of 5 base pairs in exon 4, which involves nearly the same site as the mutation reported herein; however, this mutation did not alter the reading frame.

The existence has been reported recently of at least nine isoforms of *TP53*, generated either by alternative splicing or by the presence of an internal promoter within intron 4 (none of which resembled the putative product that might be generated by the insertion found in our family with LFS) [24,25]. The role of these isoforms in the cell is not yet clear; however, it has been demonstrated that their expression is tissue-dependent, indicating that their expression is selectively regulated and that they bind differentially to endogenous *p53*-inducible promoters. However, three of these nine isoforms, denominated Δ133p53, -p53β, and -p53γ, which are generated by an internal promoter, produce mRNAs and proteins that lack the first 132 codons, involving the region where the mutation that we report herein is located. Additionally, these isoforms have been associated with breast cancer [24,25]. These isoforms begin at the ATG at position 133 (in exon 5), which at the DNA level is not affected in the case reported on here, making possible the existence of the Δ133p53 isoforms, which could participate in the carcinogenesis of the different tumors found in this family (one of them, breast cancer). In the absence of the full- length p53 protein, it is possible that expression of Δ133p53 isoforms might be favored, which might act as a dominant negative, inactivating the p53 protein generated by the wild-type allele and blocking activities such as induction of apoptosis. This could explain the pathogenicity of the insertion/duplication in this family with LFS.

## Conclusion

To our knowledge, this is the first report demonstrating this mutation associated with LFS. The functional consequence of this insertion is not known, and further analysis should be conducted to elucidate this. However, we believe that this mutation might be the cause of the LFS, because it is present in at least two affected family members. One of these developed breast cancer and died at a very young age, and in fact one half-sister of the proband also died of lung cancer at a very young age; although we did not have the opportunity to test the latter for the mutation, it is very likely that she was also a LFS carrier. It is necessary to carry out analysis of p53 mRNA and protein in this family in order to further elucidate the consequences of the mutation in the expression of p53 and the possible mechanism of carcinogenesis in carriers of the mutation.

## Consent statement

Written informed consent was obtained from the patients for publication of this case report and accompanying images. A copy of the written consent is available for review by the Editor-in-Chief of this journal.

## Competing interests

The authors declare that they have no competing interests.

## Authors' contributions

SV-M performed the clinical evaluation and genetic counseling of the family and collected data; OG-H, CT-B, and EP-C purified the DNA, performed PCR amplifications, DHPLC, and PCR products sequencing; AC-B and ED-H cloned and sequenced the exon 4 products, and LT-C and AD-G analyzed the results, and conceived of and wrote the manuscript. All authors participated in its design and coordination and helped to draft the manuscript. All authors read and approved the final manuscript.
